# Optical imaging of the breast: evaluation of deoxyhemoglobin concentration alteration in 166 patients with suspicious breast lesions

**DOI:** 10.1186/s41747-018-0038-5

**Published:** 2018-04-23

**Authors:** Antonella Petrillo, Orlando Catalano, Roberta Fusco, Salvatore Filice, Paolo Vallone, Sergio Setola, Vincenza Granata, Concetta Raiano, Franca Avino, Maurizio Di Bonito, Gerardo Botti

**Affiliations:** 1 Radiology Unit, Dipartimento di Supporto ai Percorsi Oncologici Area Diagnostica, Istituto Nazionale Tumori - IRCCS - Fondazione G. Pascale, Via Mariano Semmola, Naples, Italy; 2 Senology Surgery Unit, Dipartimento Corp-S Assistenziale e di Ricerca dei Percorsi Oncologici del Distretto Toracico, Istituto Nazionale Tumori - IRCCS - Fondazione G. Pascale, Via Mariano Semmola, Naples, Italy; 3 Pathology Unit, Dipartimento di Supporto ai Percorsi Oncologici Area Diagnostica, Istituto Nazionale Tumori - IRCCS - Fondazione G. Pascale, Via Mariano Semmola, Naples, Italy

**Keywords:** Breast cancer, Breast ultrasound, Deoxyhemoglobin concentration alteration, Mammography, Optical imaging

## Abstract

**Background:**

We investigated the performance of optical imaging evaluating deoxyhemoglobin concentration alteration (DeHCA) in breast tissues.

**Methods:**

We enrolled all consecutive patients from January 2015 to October 2016 with clinically suspicious and/or BI-RADS grade 3–5 lesions at mammography or ultrasound (US). Patients underwent optical imaging (ComfortScan) to evaluate for DeHCA. The reference standard was pathology from a surgical specimen for malignant lesions, pathology from a surgical specimen or core needle biopsy for benign lesions, and negative follow-up for contralateral negative breasts. Non-parametric statistics, receiver operating characteristic, and linear discrimination analyses were performed.

**Results:**

Of 334 enrolled patients, 168 (50%) were excluded for technical problems and 166 (50%) (median age 52 years) were analyzed totaling 331 breasts and 176 lesions. Of these, 75 were benign (median size 19 mm) and 101 malignant (median size 20 mm). The median DeHCA score in malignant lesions (0.95, interquartile range [IQR] 1.00–0.87) was higher (*p* < 0.001) than in benign lesions (0.80, IQR 0.95–0.70). Using the optimal cutoff (0.85), DeHCA score was less accurate than mammography, US, and their combination, with 78% sensitivity, 52% specificity, 40% positive predictive value (PPV), and 85% negative predictive value (NPV); using a 0.8 cutoff, sensitivity reached 93% and NPV 91%, but specificity fell to 32% and PPV to 37%. The accuracy of DeHCA score linearly combined with mammography or US was higher than that of DeHCA score alone (*p* < 0.001) and not significantly higher than that of mammography or US alone.

**Conclusions:**

DeHCA score was significantly higher in malignant than in benign lesions, but its accuracy was significantly lower than that of mammography or US. Future refinements are needed.

## Key points

- Deoxyhemoglobin concentration alteration (DeHCA) score in malignant lesions was significantly higher than that in benign lesions
- The accuracy of DeHCA score was significantly lower than that of mammography and/or ultrasound (US)
- DeHCA score accuracy linearly combined with mammography or US was significantly higher than DeHCA score alone, but not significantly higher than mammography or US alone.

## Background

Breast cancer is the most common female malignancy and the second leading cause of death from cancer among women in Europe [1]. In the United States, the estimated number of new breast cancer cases and deaths in 2017 was 318.590 and 41.070, respectively [1], with an increasing number of cases affecting young women characterized by dense breasts. Early detection of breast cancer can reduce mortality and increase survival and there is a general agreement about the correlation between tumor size at diagnosis and survival; therefore, a delay in diagnosis may negatively affect prognosis [2].

Mammography is currently the gold standard screening test for breast cancer [3]. However, it has important limitations, wherein a higher breast density, especially in younger women, can represent a major obstacle for the detection of small tumors [4, 5]. Kerlikowske et al. [6] reported that age has a considerable influence on the sensitivity of mammography, which is higher (87%) among women aged 60–69 and lower (68%) among women aged 30–39. Breast ultrasound (US) has an additional role, particularly in young women, yet has a variable reported accuracy due to its operator-dependent nature [7]. Breast magnetic resonance imaging has become an essential tool in high-risk screening, but is not suitable for large scale screening in the average-risk population [6, 8–10]. At present, no single technology allows for the optimal screening of women aged 40–69, with the combined sequence of mammography followed by US being frequently adopted in the case of negative mammography in dense breasts. In this context, new technologies for breast cancer diagnosis deserve attention, especially those that are radiation-free, either as stand-alone or for combined use with other technologies not exposing the breast to ionizing radiation.

Transillumination of the breast dates back to the 1920s [11]. However, its low sensitivity and specificity has limited its clinical use. With progress in photonic technologies and mathematic modeling of light propagation through tissues, optical imaging has evolved to a stage that allows its evaluation in a clinical setting [11, 12].

Dynamic optical imaging is based on the detection and analysis of red light transmission through the breast tissue and recording of the transitory responses of the tissue due to compression inducing changes in blood-flow volume. Such pressure stimulus results in the dynamic behavior of optical properties of the tissue, creating various dynamic profiles in regions with abnormal vascularization. Preliminary results in favor of this approach in young women were published in 2005 [13]. In 2009, dynamic optical imaging was evaluated in 46 patients, wherein the number of suspicious pixels in 12 benign lesions was significantly lower than that in 35 malignant lesions, with a sensitivity, specificity, and accuracy of 74%, 92%, and 79%, respectively [14]. Cheng et al. [15] performed dynamic optical imaging in 62 patients and obtained a sensitivity and specificity of 93% and 45%, respectively, in comparison with sensitivity and specificity values of 84% and 62% achieved with mammography. A recent work by D’Aiuto et al. [16] proposed a score system for the interpretation of dynamic optical images resulting in a sensitivity of 80% and a specificity of 87% in 113 patients preoperatively.

The aim of this study is to determine the diagnostic performance of an evolution of dynamic breast optical technology in patients with breast lesions using a score system and the combination of dynamic breast optical imaging and conventional imaging (mammography or US). The study was conducted in the context of a deoxyhemoglobin concentration alteration (DeHCA) project that assesses new applications of optical technologies in breast cancer diagnosis based on the evaluation of abnormal values of the DeHCA biomarker.

## Methods

### Study population

The study was performed at a National Cancer Center in Naples, Italy (Istituto Nazionale Tumori IRCCS Fondazione G. Pascale). The institutional review board approved the protocol (authorization number N. 718 on October 9, 2014). Informed consent was obtained from all patients. The study was performed in accordance with the current version of the Declaration of Helsinki and the International Conference on Harmonization of Good Clinical Practice Guidelines.

After a training phase on DeHCA Optical Image Processing performed on more than 150 patients, we prospectively enrolled 334 consecutive patients from January 2015 to October 2016. All patients were considered eligible when they showed suspicious breast lesions at clinical examination, and/or were evaluated as Breast Imaging Reporting and Data System (BI-RADS) score 3, 4, or 5 at mammography and/or US, were scheduled for fine needle aspiration cytology or core biopsy, and were able to be followed-up for 2 years.

Exclusion criteria were (1) the presence of a pacemaker or other devices in the chest wall; (2) an inability to keep upright immobility during the examination; (3) inflammatory skin diseases (psoriasis, eczema, etc.); (4) pregnancy or breast-feeding; (5) presence of tattoos on the breasts; (6) non-removable drilling at the nipple; (7) internal/external devices preventing from correct patient positioning; (8) particular physical shapes of the breast (e.g., too small breasts); and (9) a history of allergic reaction to silicone, the material from which the membrane used for breast compression in the optical imaging device is made. All patients underwent blind DeHCA optical image processing in the preoperative workflow. Contralateral negative breasts confirmed by a median follow-up time of 18 months (range 9–26 months) were included in the analysis to have a sufficiently large base for the evaluation of DeHCA specificity and positive predictive value (PPV).

### Mammography and US

Mammography was performed using a screen-film Senographe DMR unit (General Electric Healthcare, Milwaukee, WI, USA). Standard bilateral cranio-caudal and medio-lateral oblique mammograms were obtained. Further dedicated mammograms (i.e., magnification, spot compression, or other additional views) were obtained when necessary.

Breast US was performed using a MyLab 70 unit (Esaote, Genova, Italy), equipped with a broadband linear array transducer (5.5–12.5 MHz, generally employed at 10 MHz), which permitted a transverse resolution of 0.5 mm and a lateral resolution of 1 mm or less. Vertical, horizontal, radial, and antiradial scans of both breasts were obtained. Scanning was always extended to both axillary regions. When examining a lesion, a single focus was placed at the level of the deep aspect of the finding. The gain curve was adjusted to the depth of the lesion, while attempting to avoid artifacts. The vascular architecture of lesions identified at gray-scale B-mode imaging was investigated with color-Doppler with settings allowing for maximal sensitivity to slow flows.

Mammograms were evaluated by one of two breast radiologists with 13 and 23 years of experience, respectively. US was performed and interpreted by one of two radiologists with 15 and 23 years of experience in breast imaging, respectively. The BI-RADS categorical scoring system was used [17] to analyze images by mammography or US.

### DeHCA optical image processing

The DeHCA optical image processing is an evolution of dynamic optical imaging that effectively evaluates DeHCA values over time in response to a pressure stimulus as a biomarker to be correlated with tissue neoangiogenesis. We used the ComfortScan system (DOBI Medical International, Massachusetts, USA) for optical image acquisition.

This system is an advanced optical digital imaging device that uses high-intensity, light-emitting diodes (LEDs) and gentle external pressure to highlight areas with vascular abnormalities. The high-intensity LEDs transmit red light through the breast. If the light encounters a region with neoangiogenesis, it is absorbed or scattered differently than in other regions of the breast, as a result of differences in concentrations of oxygenated and deoxygenated hemoglobin. The system is composed of three physical assemblies, namely the C-arm assembly, the controller, and the computer system. Technical details have been previously described [13, 14].

The patients stand in front of the machine and the breast is positioned onto the panel of the C-arm assembly. The LEDs illuminate the breast and the light (wavelength 640 nm) is transmitted through the tissues and quantified on the other side by a charge coupled device (CCD) camera. In the acquisition window of the operating software, the operator can mark the region of interest placing two pointers indicating the nipple and where the lesion is supposed to be.

A soft transparent silicone membrane is placed in contact with the upper surface of the breast and then inflated under computer control during the exam. The pressure is set to 5 mmHg for the first 15 s of the scan, raised to 10 mmHg over the next 30 s (dynamic sequence), and decreased back to 5 mmHg for the final 15 s. Overall, 45 frames are acquired (5 baseline before applying pressure and 40 during the dynamic sequence). The transmitted light is detected by the CCD camera throughout the scan and recorded by the computer to generate the dynamic angiogenic signature, which is a sequence of cranial-caudal images. The analysis of this dynamic angiogenic signature identifies changes in local blood perfusion and oxygen saturation as variations in image contrast and identifies areas of pathologic interest that present an increase of both blood volume and depletion of blood oxygen with a reduction of the amount of light reaching the CCD camera [18]. This feature appears like an area of decreased intensity (dark blue) or highly decreased intensity (purple) of color contrast. ComfortScan acquisition time and map generation is equal to 90–120 s.

ComfortScan images were reviewed blindly from clinical data and from mammography and US results. The images were viewed in cine-loop modality. The temporal curve of percent intensity change versus time was displayed on a grid when the user presses on the image pixels. The integrated software classifies the temporal curves pixel by pixel and these are displayed in different chromatic scales based on the curves’ trend.

### DeHCA score

**Fig. 1:**
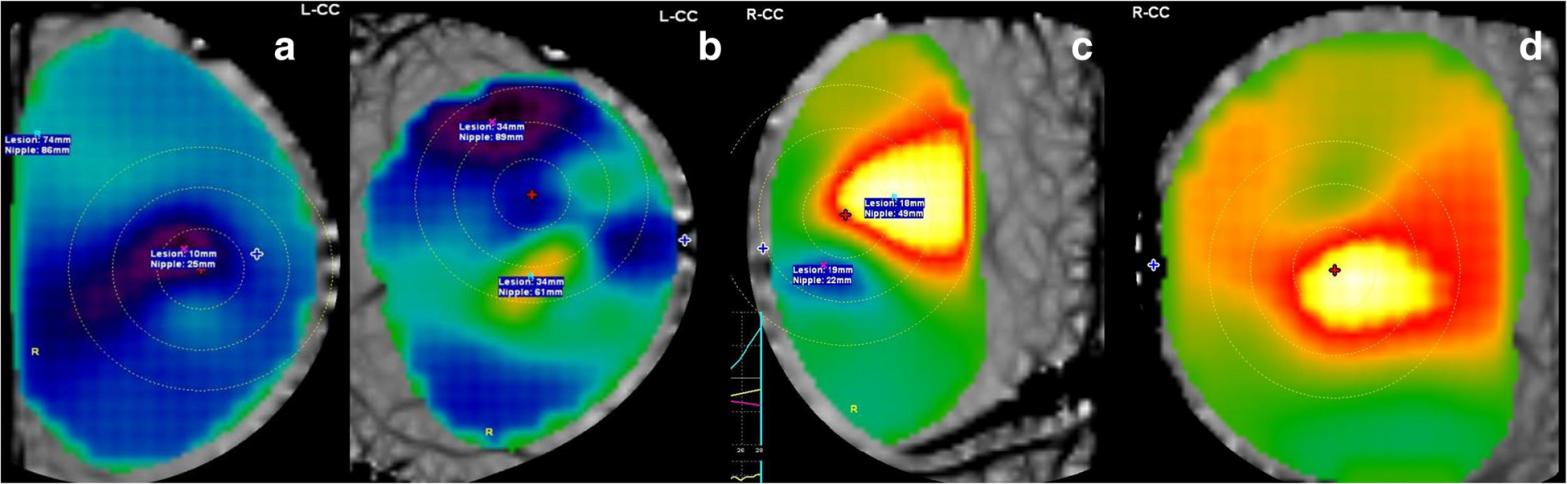
Focality description: (**a**) blue diffusion area; (**b**) area with different colors (violet present); (**c**) area with different colors (no violet); (**d**) no focality

**Fig. 2:**
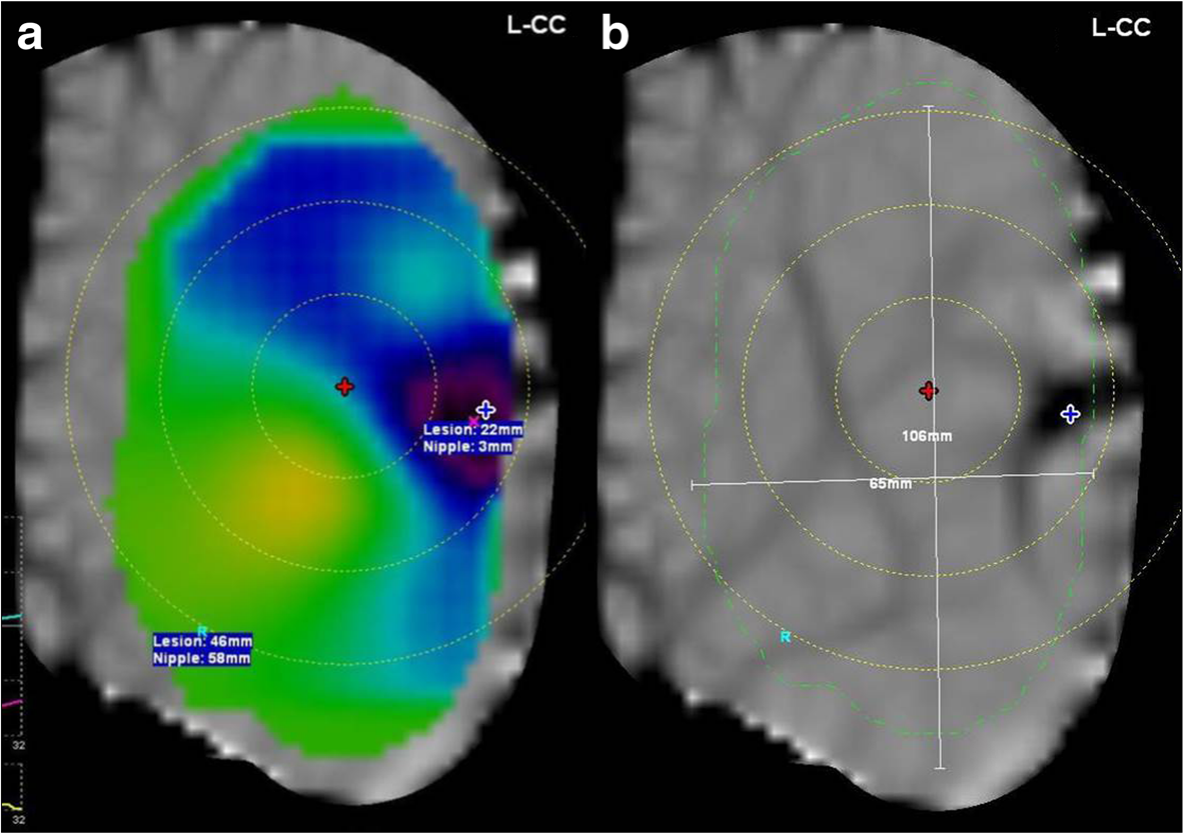
Blue nipple: (**a**) overlap between nipple (blue cross) and lesion marker; (**b**) dark spot at the nipple

**Fig. 3:**
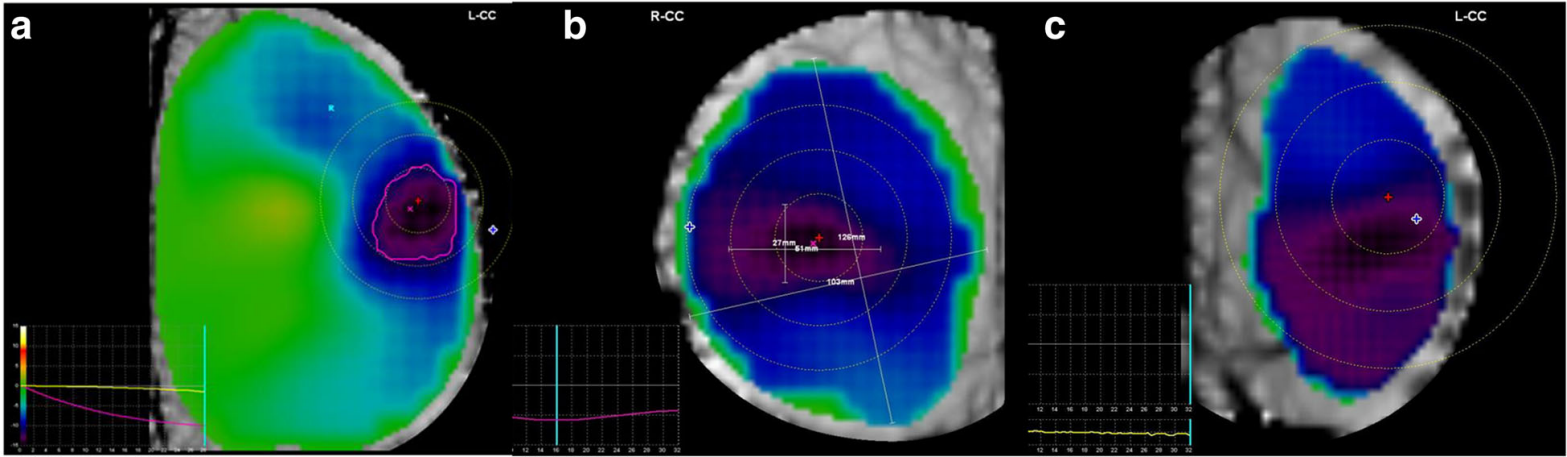
Diffusion/sharpness of the focal area: (**a**) sharp focal area; (**b**) intermediate focal area; (**c**) diffused focal area

**Fig. 4:**
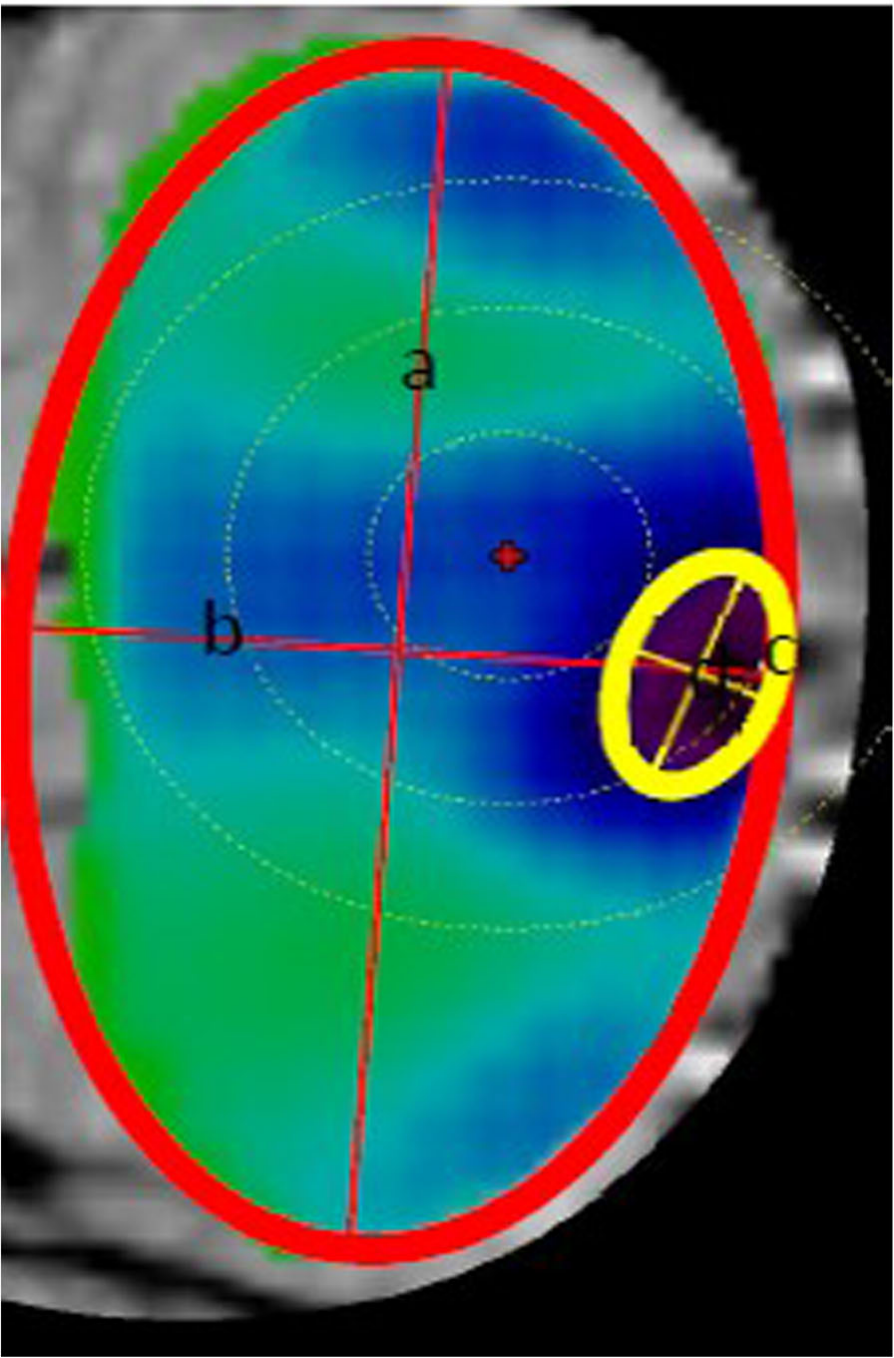
Focal area dimension: calculation of both the general colored area (modeled by the red ellipse) and the lesion area (modeled by the yellow ellipse)

**Fig. 5:**
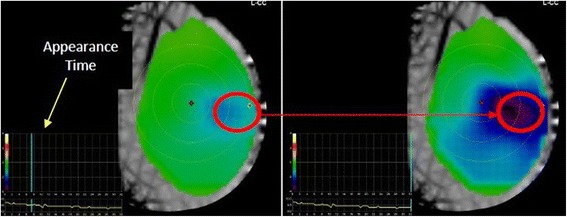
Appearance time of the focal area

**Fig. 6:**
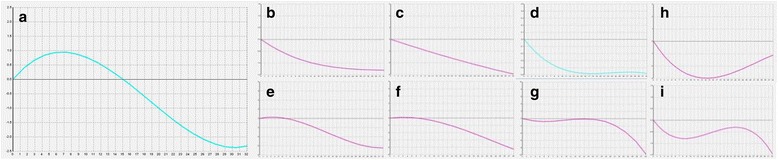
Lesion curve typology: (**a**) waving; (**b**–**d**) rapidly descending; (**e**–**g**) slowly descending; (**h**) rapidly descending with bounce; (**i**) slowly descending with final bounce

**Fig. 7:**
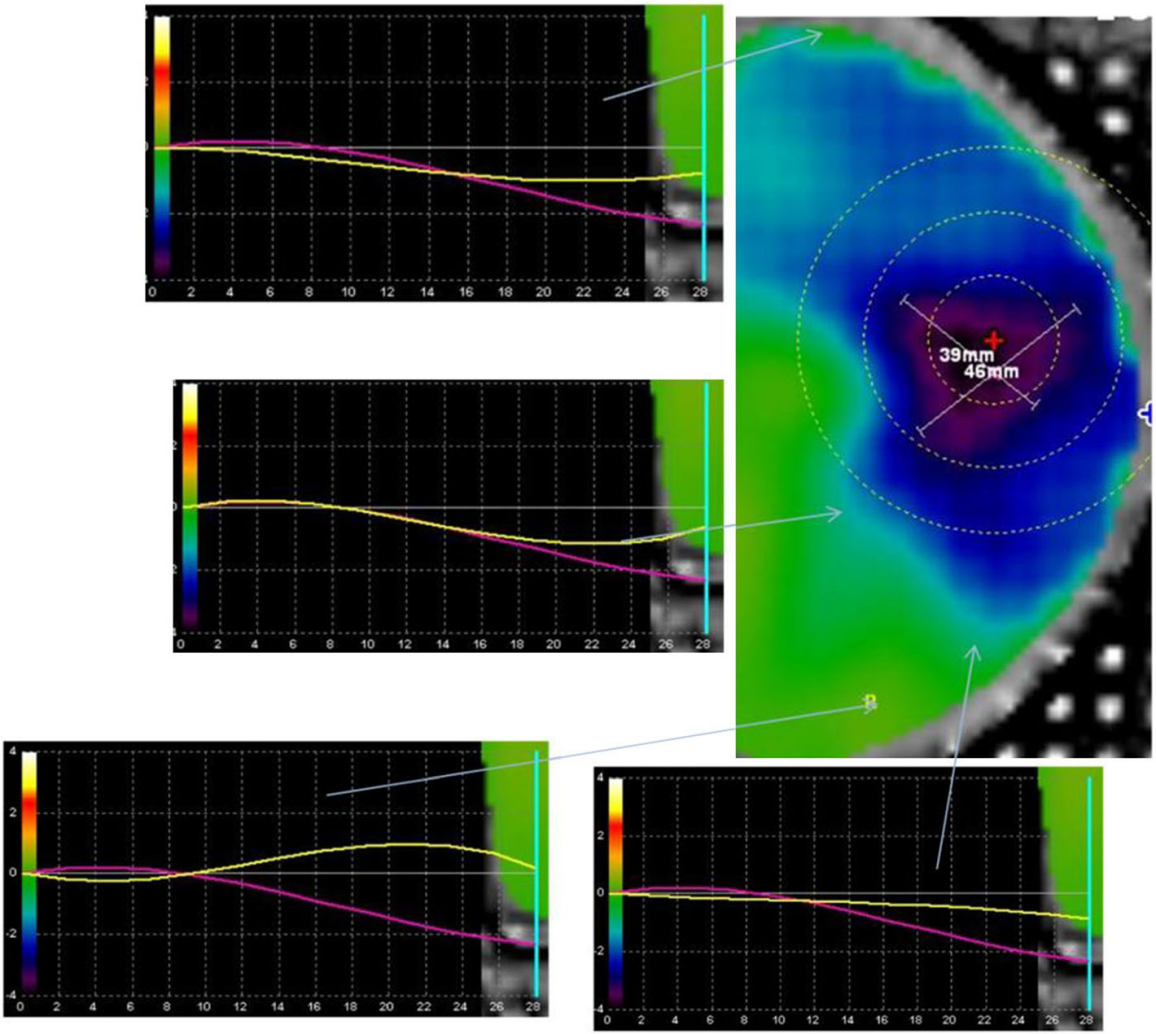
Comparison between the lesion curve and curves in other regions of the breast

**Fig. 8:**
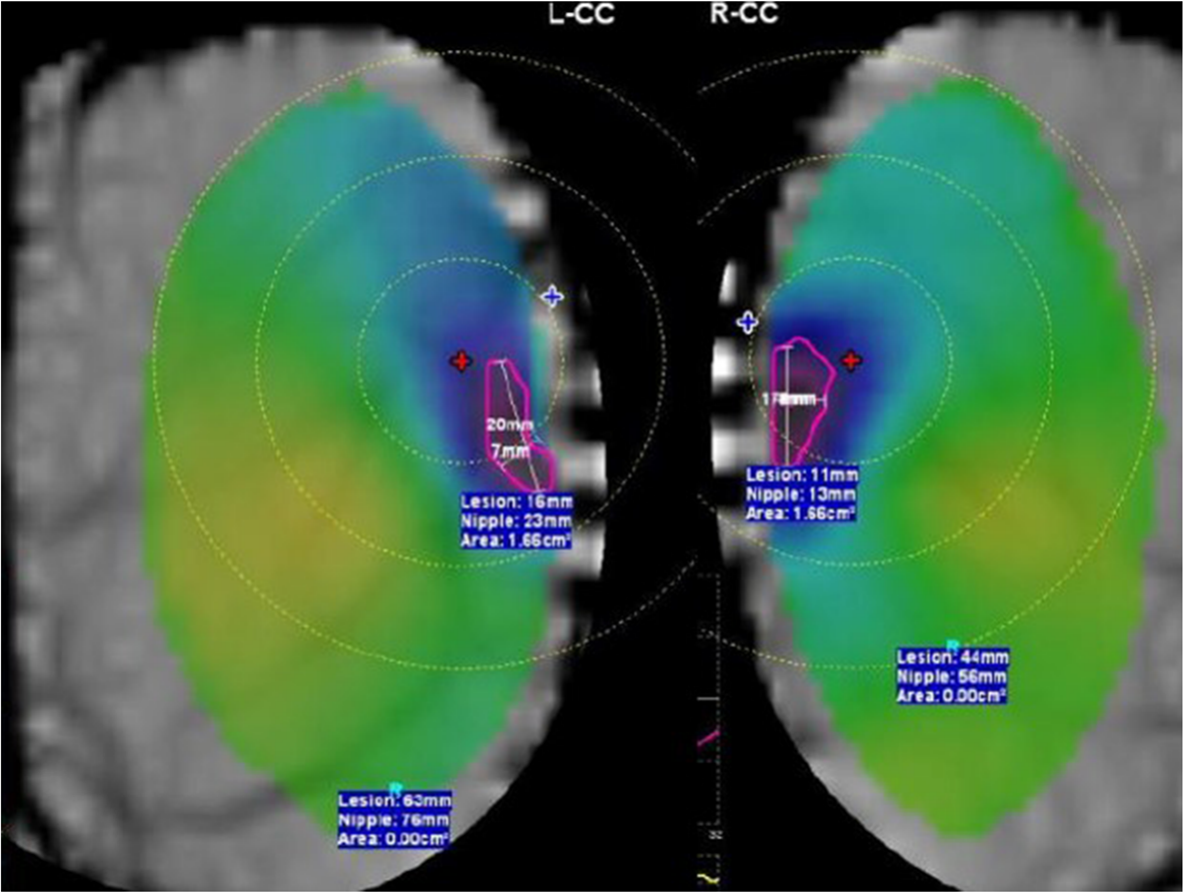
Bilaterality example: the comparison between the images of the two breasts show similar behavior

A number of parameters were evaluated to obtain the DeHCA score [18]:

1. Focality description (FOC1). Presence of ‘diffuse blue area’ throughout the area surrounding the focal point; presence of ‘different colored areas’ (yellow, green, blue, etc.) and simultaneous presence of focal points; absence of purple colored areas; no focus (Fig. 1)
2. Blue nipple (BN). Moving to the frame where the focal area shows the darkest color and positioning the lesion marker and simultaneously analyzing the curve; by moving the cursor through the subsequent pixels and identifying the curve with the most negative attenuation value; checking whether the lesion marker overlaps with the nipple marker and removing the color if there are always dark areas at the two markers (vases, typical nipples, etc.) (Fig. 2)
3. Stability/mobility of the focal area related to the epicenter (EPI)*.* Assesses whether, in respect to the marked lesion marker, the focal epicenter moves when the black area is formed or before its disappearance
4. Sharpness of the focal area (FOC2). Characteristics of the focal area with the maximum attenuation, using three degrees, as follows: (1) sharp focal area similar to a well-defined atoll sharp and compact dark spot inside the focal area; violet contour closely following the edges of the black spot, almost drawn; (2) intermediate focal area similar to an atoll, with less sharp borders than the previous case, compact black spot with pixel effect, violet spot not closely surrounding the edges of the black spot; (3) non-compact focal area, with the black spot loosely defined, black pixels spreading within the violet area, and the black/violet area expanding on a large portion of the overall area (Fig. 3)
5. Focal area size (AR)*.* Ratio between lesion area and ellipse area calculated by the formula AR = (a/2) × (b/2) × π (Fig. 4)
6. Maximum attenuation. Referring to the frame with maximum attenuation and visualizing the attenuation value positioning exactly on the lesion marker
7. Time of appearance (TEMP). Observing the previously positioned lesion marker, detecting the frame where the focal area starts forming near the marker in terms of a well-defined attenuation area; the color might start appearing light blue and become darker (Fig. 5)
8. Lesion curve typology. Classifying the lesion marker curve as one of the following: waving (integral value equal to null); rapidly descending (rapidly moves away); slowly descending; rapidly descending with bounce (where the final value varies more than 20% from the value of maximum attenuation); slowly descending with final bounce (Fig. 6)
9. Presence of similar/dissimilar areas (SIM). Comparison between the lesion curve and curves in other regions of the breast to evaluate the presence of similar behaviors inside the breast (Fig. 7)
10. Bilaterality (BIL). Simultaneous presence of the following conditions: (1) the same maximum symmetry for both breast; (2) the same maximum attenuation (differentiation less 30%); (3) the same curve type (Fig. 8)

The DeHCA score was calculated using the previous parameters with the formula reported in Table 1 (DeHCA score = FOC1 × BN × EPI × FOC2 × AR × BIL × SIM × TEMP) in order to evaluate the probability of the presence of a breast cancer lesion. DeHCA score reporting time was 12 min (median value).

**Table 1:** Parameters for DeHCA score calculation

Parameter	State	Score	Acronym
Focality description	Absent	0	FOC1
	Present	1	
Blue nipple	Yes	0	BN
	No	1	
Stability/mobility of focality (Epicenter)	Mobile	0	EPI
	Stable	1	
Diffusion/sharpness of the focal area	Diffuse	0	FOC2
	Intermediate	0.95	
	Sharp	1	
Focal area/total area	Area > 20%	0	AR
	10% < Area < 20%	0.85	
	Area < 10%	1	
Bilaterality	Yes	0	BIL
	No	1	
Similarity	Yes	0.8	SIM
	No	1	
Appearance time (t)	t > 15	0.85	TEMP
	10 < t ≤ 15	0.92	
	t ≤ 10	1	
DeHCA score = FOC1 × BN × EPI × FOC2 × AR × BIL × SIM × TEMP	0 < score < 0.8 (low risk)		
	0.8 < score < 0.9 (intermediate risk)		
	0.9 < score < 1 (high risk)		

*AR* focal area size, *BIL* bilaterality, *BN* Blue nipple, *EPI* stability/mobility of the focal area related to the epicenter, *FOC1* focality description, *FOC2* sharpness of the focal area, *SIM* presence of similar/dissimilar areas, *TEMP* time of appearance

### Reference standard and pathological methods

Each suspicious finding at mammography and/or US underwent pathology analysis. The reference standard was pathology from a surgical specimen for malignant lesions, pathology from a surgical specimen or core needle biopsy for benign lesions, and negative follow-up for contralateral negative breasts. Each patient work-up was decided following the joint decision of a multidisciplinary team, including at least one radiologist and one surgeon. Tumor and nodal stage were classified according to the system implemented by the American Joint Committee on Cancer staging [19]. The intensity, extent, and subcellular distribution of estrogen receptor (ER), progesterone receptor (PR), human epidermal growth factor receptor 2 (HER2), and Ki67 were evaluated as previously described [20]. The cutoff used to distinguish positive from negative cases was ≥ 1% for ER/PR ratio. Scores of 0 or 1+ were considered negative for HER2 expression, 2+ and 3+ scores were positive. In case of HER2 score equal to 2+, fluorescence in situ hybridization (FISH) amplification was performed and HER2 expression was considered as positive HER2 amplification when the FISH ratio was higher than 2.2 or the HER2 gene copy greater than 6.0; as equivocal HER2 amplification at a FISH ratio of 1.8–2.2 or HER2 gene copy of 4.0–6.0; and as negative HER2 amplification when the FISH ratio was lower than 1.8 or HER2 gene copy less than 4.0. The percentage of positive cells per case for proliferative index Ki67 was scored according to two different groups – group 1, < 15% (low proliferative activity, negative cases); group 2, ≥ 15% (high proliferative activity, positive cases). Ductal carcinoma in situ and invasive cancer tumors were counted as malignant lesions. All other results, including lobular carcinoma in situ, fibroadenoma, ductal hyperplasia, dysplasia, cysts, and phyllodes tumors, were considered non-malignant lesions.

### Statistical analysis

The DeHCA score performance was evaluated by using both the optimal cutoff value (0.85) and also the 0.80 value (to maximise sensitivity). Continues variables were reported as median and 75th and 25th percentile (interquartile range, IQR). Receiver operating characteristic (ROC) analysis was used to define the optimal cutoff maximizing the Youden index. Sensitivity, specificity, PPV, negative predictive value (NPV), and accuracy were calculated for mammography, US, combined mammography and US, and DeHCA score. For inter-group comparisons, we used the Mann Whitney *U* test for continuous variables and χ^2^ test for categorical variables. Spearman’s correlation coefficient was also calculated to compare DeHCA score and BI-RADS score with pathological findings. Additionally, we assessed the diagnostic accuracy for a linear classifier that combines DeHCA score with mammography ([a × DeHCA score] + [b × mammography BI-RADS category] > constant) and for a linear classifier that combines DeHCA score with US mammography ([a × DeHCA score] + [b × US BI-RADS category] > constant). The linear classifier of the Statistics and Machine Learning Toolbox of Matlab R2007 individuates the coefficient of linear combination and the constant; when the weighted sum exceeds the constant, the classifier returns a test positive case. McNemar test was used to compare the diagnostic performance in terms of sensitivity and specificity. A *p* value of less than 0.05 was considered significant. Calculations were performed using the Statistics and Machine Learning Toolbox of Matlab R2007a (MathWorks, Natick, USA).

## Results

Of 334 enrolled patients, 168 patients (50%) were excluded for technical problems, 94 patients (28%) for technical failure at optical image processing, 73 (22%) due to having too small or large breasts, and 1 for having breasts that were too dense. Therefore, 166 (50%) patients entered the analysis, with a median age of 52 years (IQR 64–44 years). A total of 176 lesions and 331 breasts were investigated (one breast for one patient was eliminated for absence of cytological characterization in the work-up).

Overall, at pathology, 75 lesions were benign (median size 19 mm, IQR 24–11 mm) and 101 were malignant (median size 20 mm, IQR 26–16 mm), and a total of 155 breasts had negative follow-up at conventional imaging (Table 2). The distribution of pathology prognostic factors is provided in Table 3. For seven patients with Herceptest status equal to 2+, FISH analysis was performed, returning three positive cases and four negative cases. There were significant differences between groups in terms of age and positive versus negative ER/PR expression, while there were no significant differences between groups in terms of grading, Herceptest status, and for positive and negative Ki67. BI-RADS score distribution was reported in Table 4.

**Table 2:** Histotype of breast lesions in the studied population

Disease	Subtype	Number of breasts	Percentage
Non-malignant	Adenosis	10	13.3
	Dysplasia	14	18.7
	Fibroadenoma	30	40.0
	Inflammation	9	12.0
	Ductal hyperplasia	9	12.0
	Benign phyllodes	1	1.3
	Lobular carcinoma in situ	2	2.7
	Total	75	100
Malignant	Ductal carcinoma in situ	3	3.0
	Invasive ductal carcinoma	76	75.2
	Invasive cribriform carcinoma	1	1.0
	Invasive lobular carcinoma	13	12.9
	Invasive mucinous carcinoma	1	1.0
	Invasive tubular carcinoma	5	5.0
	Carcinomatous mastitis	1	1.0
	Paget disease	1	1.0
	Total	101	100

**Table 3:** Association between DeHCA score and prognostic factors

Patients’ DeHCA score	*<* 0.8		≥ 0.8		*P* ^a^
	Number	Percentage	Number	Percentage	
Age					< 0.001
≤ 40	23	47.9	25	52.1	
40–49	34	33.3	68	66.7	
50–59	8	10.5	68	89.5	
≥ 60	10	9.5	95	90.5	
Tumor grading					0.883
I	1	20.0	4	80.0	
II	3	5.2	55	94.8	
III	1	8.3	11	91.7	
Herceptest					0.470
Positive	0	0	13	100	
Negative	7	11.7	57	88.3	
Receptor status (ER/PR)					0.031
Positive	2	4	48	96	
Negative	4	26.7	11	73.3	
Ki67					0.082
Positive	0	0	28	100	
Negative	7	14.9	40	85.1	

^a^χ^2^ test with Yates’ correction

**Table 4:** BI-RADS diagnostic categories distribution in the studied population

	Mammography BI-RADS	US BI-RADS	Mammography/US BI-RADS^a^
Non-malignant lesions (n = 75)			
BI-RADS 0	16	2	
BI-RADS 1	7	0	
BI-RADS 2	4	3	
BI-RADS 3	22	40	37
BI-RADS 4	21	22	29
BI-RADS 5	3	6	7
Malignant lesions (n = 101)			
BI-RADS 0	14	1	
BI-RADS 1	6	0	
BI-RADS 2	0	1	
BI-RADS 3	3	3	3
BI-RADS 4	19	20	15
BI-RADS 5	59	76	83
Negative breasts (n = 155)			
BI-RADS 0	47	31	27
BI-RADS 1	105	115	118
BI-RADS 2	2	5	6
BI-RADS 3	0	4	4
Total	331	331	331

^a^The higher BI-RADS value between mammography and US

**Fig. 9:**
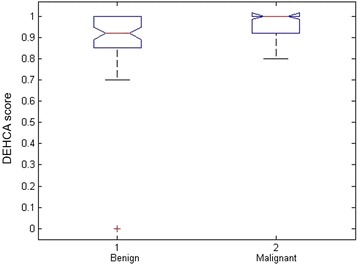
Boxplot of DeHCA score to differentiate malignant versus non-malignant lesions

The DeHCA score of malignant lesions (median 0.95, IQR 1.0–0.87) was significantly higher than that of benign lesions (median 0.8, IQR 0.95–0.7) (*p* < 0.001, Mann Whitney *U* test, see also boxplot of DeHCA score in Fig. 9). Table 5 reports the Spearman’s correlation coefficients to compare the DeHCA and BI-RADS scores with pathological findings. No correlations were observed.

**Table 5:** Spearman’s correlation coefficients

		DeHCA score	Mammography BI-RADS	US BI-RADS	ER	PR	Ki67	Herceptest
DeHCAscore	Correlation coefficient	1.0	0.221^**^	0.152^**^	0.072	−0.093	0.094	0.026
	*p* value		0.000	0.006	0.565	0.502	0.445	0.893
Mammography BI-RADS	Correlation coefficient	0.229^**^	1.0	0.629^**^	0.046	0.105	0.191	−0.005
	*p* value	0.000		0.000	0.755	0.412	0.091	0.971
US BI-RADS	Correlation coefficient	0.159^**^	0.629^**^	1.0	−0.169	−0.021	− 0.149	0.075
	*p* value	0.006	0.0		0.256	0.943	0.228	0.532
ER	Correlation coefficient	0.071	0.043	−0.162	1.0	0.431^**^	0.152	0.051
	*p* value	0.565	0.755	0.267		0.001	0.244	0.681
PR	Correlation coefficient	−0.091	0.105	−0.024	0.411^**^	1.0	−0.132	−0.291^*^
	*p* value	0.512	0.411	0.862	0.001		0.433	0.020
Ki67	Correlation coefficient	0.092	0.191	−0.143	0.153	−0.123	1.0	0.274^*^
	*p* value	0.442	0.093	0.228	0.243	0.432		0.016
Herceptest	Correlation coefficient	0.022	−0.005	0.079	0.055	−0.291^*^	0.271^*^	1.0
	*p* value	0.891	1.0	0.534	0.682	0.022	0.016	

*US* ultrasound, *ER* estrogen receptor, *PR* progesterone receptor

^*^Significant correlation, *p* < 0.05

^**^Significant correlation, *p* < 0.01

Table 6 reports the diagnostic performance of mammography, US, mammography/US, and DeHCA score in the detection of breast abnormalities, including benign and malignant cases versus negative cases (no abnormalities). Table 7 reports the detection of malignant lesions.

**Table 6:** Diagnostic performance for discriminating breast diseases versus negative cases for mammography BI-RADS score, US BI-RADS score, mammography/US BI-RADS score, and DeHCA score

	Area under the curve	Sensitivity	Specificity	Positive predictive value	Negative predictive value	Accuracy
Mammography BI-RADS	0.81	0.72	0.97	0.96	0.77	0.84
US BI-RADS	0.96	0.94	0.96	0.96	0.94	0.95
Mammography/US BI-RADS	0.98	0.96	0.95	0.95	0.96	0.96
DeHCA score (optimal cutoff = 0.85)	0.55	0.62	0.47	0.54	0.56	0.55
DeHCA score (cutoff = 0.80)	0.55	0.81	0.28	0.59	0.60	0.54

**Table 7:** Diagnostic performance for detecting malignant lesions for mammography BI-RADS score, US BI-RADS score, mammography/US BI-RADS score, and DeHCA score

	Area under the curve	Sensitivity	Specificity	Positive predictive value	Negative predictive value	Accuracy
Mammography BI-RADS	0.83	0.77	0.89	0.74	0.90	0.85
US BI-RADS	0.96	0.96	0.87	0.76	0.98	0.90
Mammography/US BI-RADS	0.97	0.98	0.84	0.72	0.99	0.88
DeHCA score (optimal cutoff = 0.85)	0.65	0.78	0.52	0.40	0.85	0.60
DeHCA score (cutoff = 0.80)	0.65	0.93	0.32	0.37	0.91	0.50

**Fig. 10:**
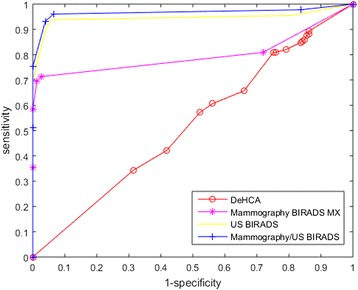
ROC curve in the discrimination of breast diseases including non-malignant and malignant cases versus negative ones

**Fig. 11:**
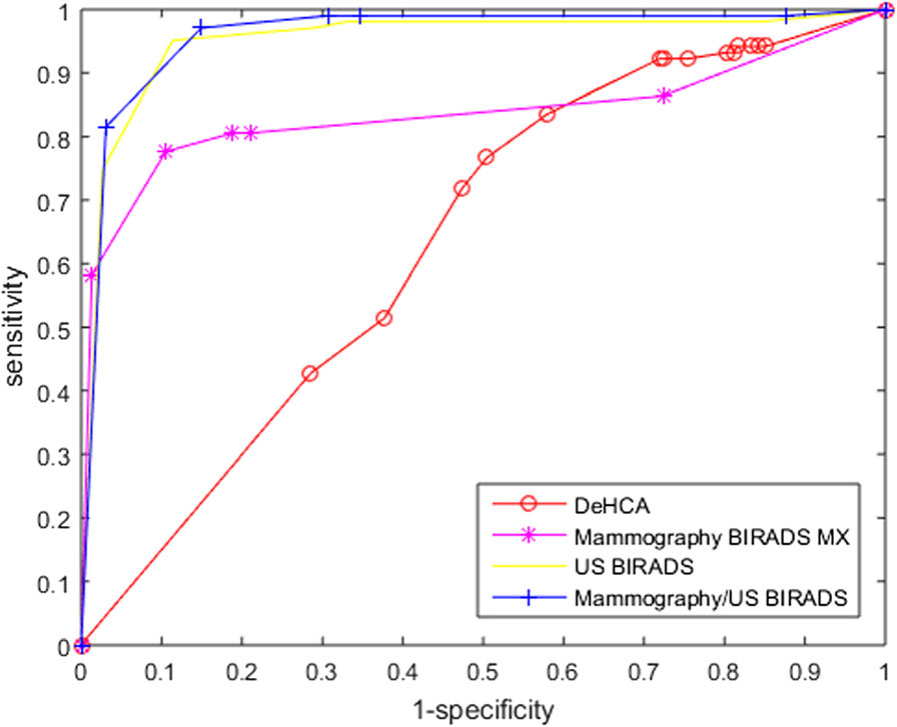
ROC curve to discriminate malignant lesions

**Fig. 12:**
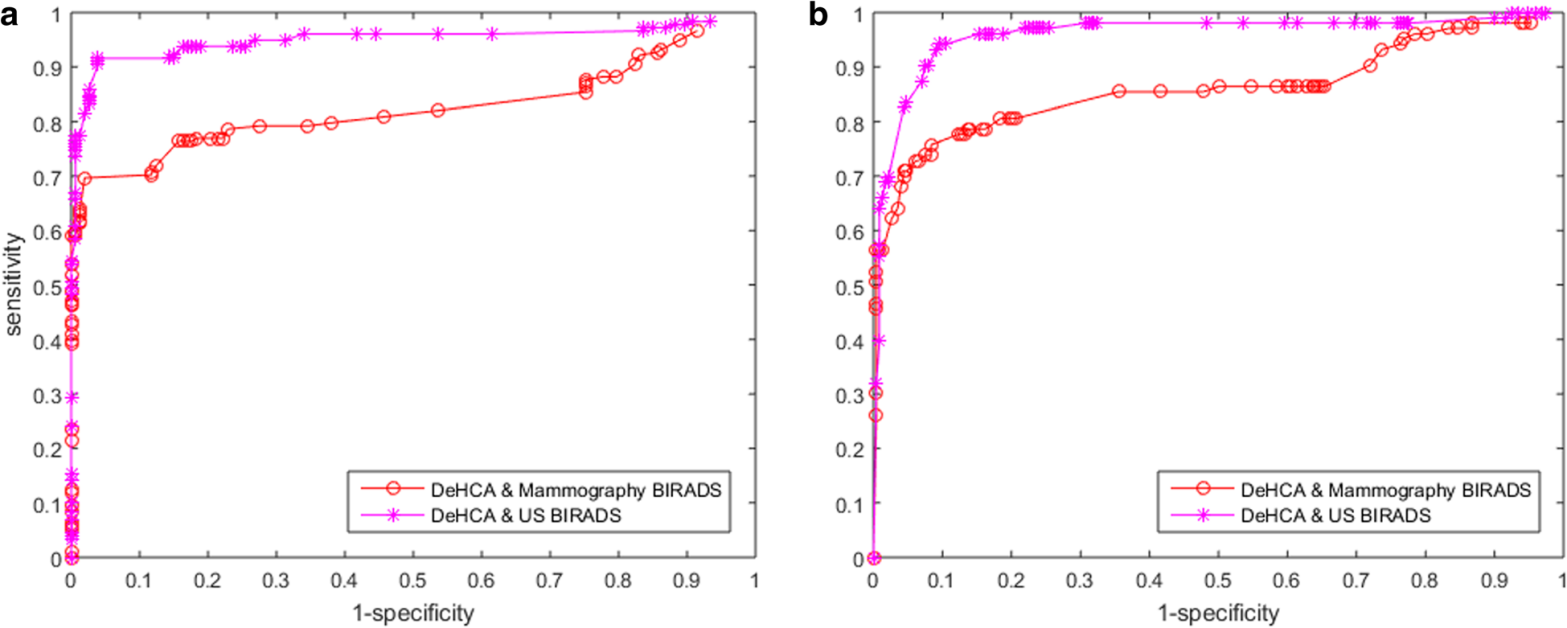
ROC curves for both linear classifiers (DeHCA score and mammography and DeHCA score and US) to (**a**) discriminate breast diseases versus negative cases and (**b**) differentiate malignant from non-malignant breast lesions

Figures 10 and 11 show the ROC curves for both classification analyses. DeHCA optical image processing showed a lower accuracy compared to mammography, US, and their combination using either the optimal cutoff value (0.85) or the cutoff of 0.8. Table 8 reports the diagnostic performance of the linear classifier that combines DeHCA score with mammography and of the linear classifier that combines DeHCA score with US. Figure 12 shows ROC curves for both linear classifiers to (1) discriminate breast diseases from negative cases and (2) detect malignant lesions.

**Table 8:** Diagnostic performance of DeHCA score plus mammography linear combination and of DeHCA score plus US linear combination

	Area under the curve	Sensitivity	Specificity	Positive predictive value	Negative predictive value	Accuracy
To discriminate breast disease versus negative cases						
DeHCA plus mammography	0.78	0.72	0.96	0.94	0.78	0.84
DeHCA plus US	0.93	0.94	0.96	0.96	0.94	0.95
To detect malignant lesions						
DeHCA plus mammography	0.73	0.93	0.82	0.89	0.87	0.73
DeHCA plus US	0.96	0.92	0.81	0.98	0.93	0.96

The linear combination of DeHCA score and mammography and the linear combination of DeHCA score and US showed a significantly higher accuracy than DeHCA score alone (*p* < 0.001, McNemar test) both to discriminate breast diseases from negative cases and to distinguish malignant lesions; the increase in accuracy was not significant compared to mammography alone or US alone (DeHCA score plus mammography versus mammography alone, *p* = 0.421; DeHCA score plus US versus US alone, *p* = 0.302; both McNemar test). Moreover, DeHCA score combined linearly to US in the detection of malignant lesions showed a higher accuracy (93%), although not statistically significant, than mammography alone (85%) and a higher sensitivity (96%) than mammography alone (77%) (*p* = 0.09, McNemar test).

**Fig. 13:**
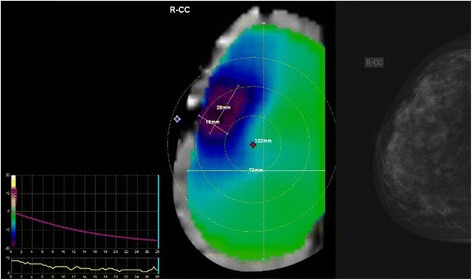
Mammography appearance with prevalent, fibroglandular component most represented in the superior external quadrant, with consequent limited diagnostic definition. There is no obvious focal opacity. Instead, the DeHCA score was equal to 0.85. Final diagnosis: invasive ductal carcinoma

**Fig. 14:**
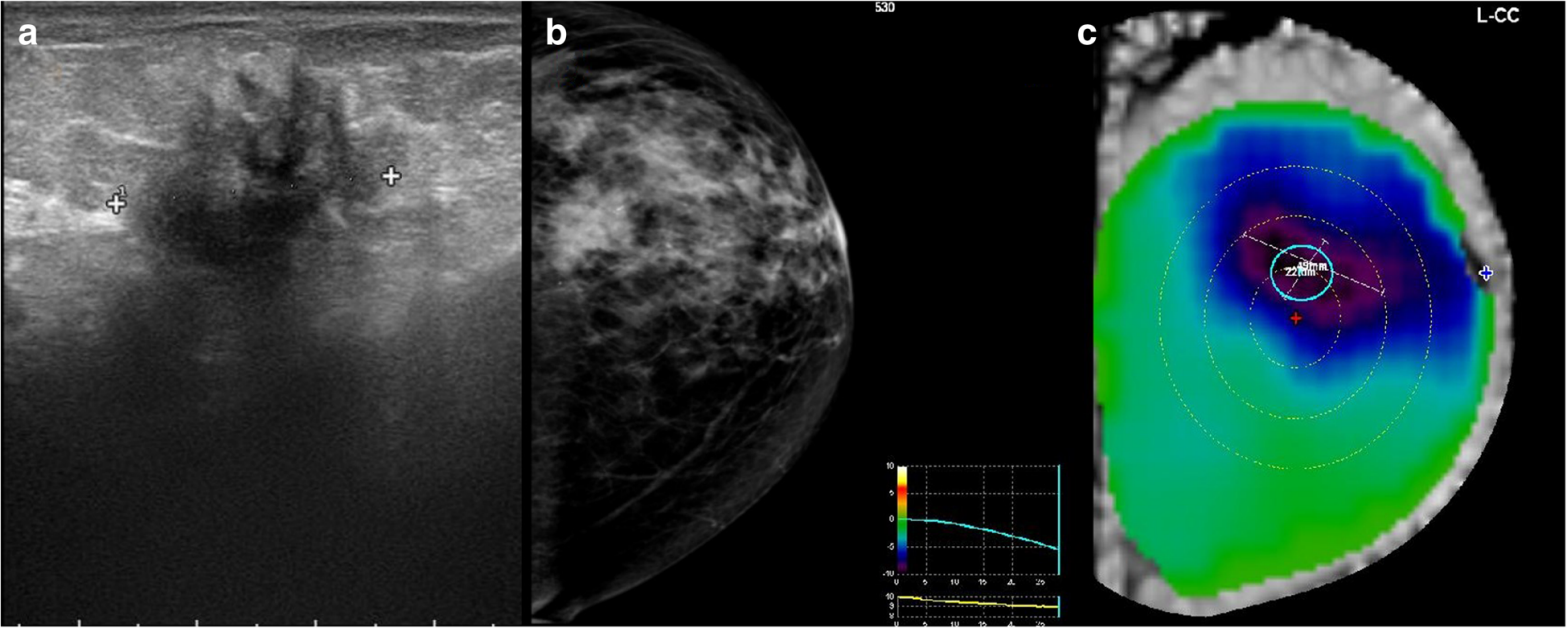
Mammography (**a**) shows a heterogeneous finding with irregular edges of approximately 30 cm in diameter. DeHCA score (**b**) was equal to 0.92. At US (**c**), in the outer quadrant of the left breast, a hypoechoic nodule with irregular borders is visible, measuring 12 × 6 mm, with internal dot-like calcifications. Final diagnosis: invasive ductal carcinoma

A false negative at mammography was shown to be a true positive at DeHCA optical image processing (score 0.85) (Fig. 13). With US, a hypoechoic nodule was observed in the outer quadrant of the left breast with irregular borders of 12 × 6 mm with calcifications inside (Fig. 14). Mammography showed a heterogeneous hyperdense lesion with irregular borders of approximately 30 cm in diameter. DeHCA optical image processing gave a true positive result (score 0.92).

## Discussion

Dynamic optical breast imaging may identify breast tumors by detecting changes in local blood perfusion and oxygen saturation due to neoangiogenesis. The dynamic optical breast imaging has numerous potential advantages, including the use of non-ionizing, low-energy light radiation, continuous data acquisition for real-time monitoring, and low cost. To date, few papers investigating the use of the dynamic optical breast imaging for the early detection of breast cancer are available.

Athanasiou et al. [13] reported the results in a series of 72 patients with BI-RADS score 4–5 breast lesions. The diagnostic accuracy reached by the optical technology applied to BI-RADS score 4 or 5 breast lesions was 73% sensitivity and 38% specificity. False negatives were mostly related to small lesions (< 10 mm), while false positives were mainly benign proliferative lesions. The interpretation of optical imaging was based on the analysis of the presence of early, focal, intense blue color in the area of interest, the pixel intensity of the blue areas, and the type of temporal signature of dynamic curves. A numeric level of suspicious score was calculated based on all these elements and a score more than 5 was considered a suspect. However, the criteria of imaging interpretation were not clearly defined and there was a high interobserver variability in the determination of the score.

Frattini et al. [21] performed a prospective analysis of 617 young women evaluating the diagnostic accuracy of the combined use of optical technology and US, and found a sensitivity of 97% and specificity of 87%, a net increase in accuracy compared to US alone (sensitivity 74%, specificity 70%). Fournier et al. [14] evaluated the dynamic optical breast imaging system in 47 BI-RADS score 3–5 breast lesions. A significant difference in numbers of suspect pixels between 12 benign lesions and 35 malignant lesions was observed; the ROC curve showed an optimal cutoff for the number of pixels equal to 2050, which determined a sensitivity of 26/35 (74%) and a specificity of 11/12 (92%). Of note, 6 out of 9 malignant lesions missed by dynamic optical breast imaging had been classified BI-RADS score 5 by mammography. In view of that, the authors stated that mammography and optical imaging should be considered complementary, as they describe different physiological properties of tissues.

Considering that, to date, breast diagnosis relies on the combined use of complementary technologies, the aim of this study is to determine the diagnostic performance of an evolution of dynamic breast optical technology in patients with breast lesions using a score system and the combination of dynamic breast optical imaging and conventional imaging (mammography or US).

We reported a statistical significant difference in DeHCA score median value for benign versus malignant breast lesions (*p* < 0.001, Mann Whitney *U* test) but no correlations were observed between DeHCA and BI-RADS scores as well as with the pathological findings. The measurement of the DeHCA biomarker made using Comfortscan images showed a lower accuracy than mammography, US, and their combination using the optimal cutoff (0.85) obtained by ROC analysis, with 78% sensitivity, 52% specificity, 40% PPV, and 85% NPV; using a threshold of 0.8, sensitivity reached 93% and NPV 91%, but specificity fell to only 32% and PPV to only 37%.

The linear combination of DeHCA score and mammography and the linear combination of DeHCA score and US showed a significantly higher accuracy than DeHCA score based on Comfortscan alone; the increase in accuracy was not significant compared to mammography alone or US alone. Moreover, DeHCA score combined linearly to US in the detection of malignant lesions showed a higher accuracy (93%) than mammography alone (85%) and a higher sensitivity (96%) than mammography alone (77%; *p* = 0.09, McNemar test).

In this perspective, DeHCA optical image processing may play a role in breast assessment in combination with US, although the additional diagnostic contribution of DeHCA optical image processing to that of US alone deserves further investigation.

There are some limitations to our study that must be considered. The high exclusion percentage for technical problems (50%) is as a limitation of the current optical imaging procedure investigated herein. However, technological improvements are being continually developed to reduce the number of eliminations. Particularly in the early phase of the study, we had several technical failures mainly due to the use of ComfortScan hardware, which is an evolving digital optical scanner. Breast imaging was acquired only in cranio-caudal projection, which may cause an underdetection of lesions localized in the axillary pilaster and/or mammary sulcus. Moreover, DeHCA optical image processing was not feasible in some women with very small, firm breasts that could not be properly illuminated as well as in women previously submitted to surgery and/or bioptic procedures due to the presence of residual edema and extravasation. The possible limiting role of factors like inflammatory breast conditions, skin breast tattoos, menstrual cycle phases, and vasculitis in the appropriate DeHCA optical image processing should be addressed in appropriate trials. The overall disease prevalence in our series, and the high number of malignant versus benign lesions among the positive cases, is not truly representative of the female population normally investigated in a screening breast imaging center. This was due to the second level nature of our cancer center and to the need to have a pathological standard reference available for the study purposes. In addition, not all cases had both mammography and US available. Finally, it must also be considered that we used film-screen mammography, which is no longer considered the state-of-the art technique, despite still being utilized in many screening programs.

In conclusion, in our experience, optical imaging provided a DeHCA score in malignant lesions that was significantly higher than that of benign lesions. However, using the optimal cutoff (0.85) or a threshold of 0.8 for DeHCA score, accuracy remained significantly lower than mammography or US alone, not allowing for a stand-alone use of the current status of this technology in breast cancer diagnosis. The linear combination of DeHCA score and mammography and of DeHCA score and US significantly increased the diagnostic performance accuracy with respect to DeHCA score alone. Future technological refinements are needed to make this technology ready for clinical practice. In this possible future scenario, DeHCA technology in combination with US might be an interesting perspective.

## Abbreviations

AR: focal area size
BIL: bilaterality
BN: blue nipple
CCD: charge coupled device
DeHCA: deoxyhemoglobin concentration alteration
EPI: stability/mobility of the focal area related to the epicenter
ER: estrogen receptor
FISH: fluorescence in situ hybridization
FOC1: focality description
FOC2: sharpness of the focal area
HER2: human epidermal growth factor receptor 2
IQR: interquartile range
LED: light-emitting diode
NPV: negative predictive value
PPV: positive predictive value
PR: progesterone receptor
ROC: receiver operating characteristic
SIM: presence of similar/dissimilar areas
TEMP: time of appearance
US: ultrasound
